# Modelling of viral load dynamics and CD4 cell count progression in an antiretroviral naive cohort: using a joint linear mixed and multistate Markov model

**DOI:** 10.1186/s12879-020-04972-1

**Published:** 2020-03-26

**Authors:** Zelalem G. Dessie, Temesgen Zewotir, Henry Mwambi, Delia North

**Affiliations:** 1 School of Mathematics, Statistics and Computer Science, University of KwaZulu-Natal, Durban, South Africa; 2 College of Science, Bahir Dar University, Bahir Dar, Ethiopia

**Keywords:** Factor analysis, Latent variables, Mixed effect model, Multistate model, Quality of life

## Abstract

**Background:**

Patients infected with HIV may experience a succession of clinical stages before the disease diagnosis and their health status may be followed-up by tracking disease biomarkers. In this study, we present a joint multistate model for predicting the clinical progression of HIV infection which takes into account the viral load and CD4 count biomarkers.

**Methods:**

The data is from an ongoing prospective cohort study conducted among antiretroviral treatment (ART) naïve HIV-infected women in the province of KwaZulu-Natal, South Africa. We presented a joint model that consists of two related submodels: a Markov multistate model for CD4 cell count transitions and a linear mixed effect model for longitudinal viral load dynamics.

**Results:**

Viral load dynamics significantly affect the transition intensities of HIV/AIDS disease progression. The analysis also showed that patients with relatively high educational levels (β = − 0.004; 95% confidence interval [CI]:-0.207, − 0.064), high RBC indices scores (β = − 0.01; 95%CI:-0.017, − 0.002) and high physical health scores (β = − 0.001; 95%CI:-0.026, − 0.003) were significantly were associated with a lower rate of viral load increase over time. Patients with TB co-infection (β = 0.002; 95%CI:0.001, 0.004), having many sex partners (β = 0.007; 95%CI:0.003, 0.011), being younger age (β = 0.008; 95%CI:0.003, 0.012) and high liver abnormality scores (β = 0.004; 95%CI:0.001, 0.01) were associated with a higher rate of viral load increase over time. Moreover, patients with many sex partners (β = − 0.61; 95%CI:-0.94, − 0.28) and with a high liver abnormality score (β = − 0.17; 95%CI:-0.30, − 0.05) showed significantly reduced intensities of immunological recovery transitions. Furthermore, a high weight, high education levels, high QoL scores, high RBC parameters and being of middle age significantly increased the intensities of immunological recovery transitions.

**Conclusion:**

Overall, from a clinical perspective, QoL measurement items, being of a younger age, clinical attributes, marital status, and educational status are associated with the current state of the patient, and are an important contributing factor to extend survival of the patients and guide clinical interventions. From a methodological perspective, it can be concluded that a joint multistate model approach provides wide-ranging information about the progression and assists to provide specific dynamic predictions and increasingly precise knowledge of diseases.

## Background

HIV infection is one of the leading causes of death from infectious diseases globally and remains a serious *global public health issue* [1, 2]. AIDS, the last progress stage of HIV infection, leads to severe damage to the body’s immune system [3]. The progression of HIV/AIDS is highly variable between populations and individuals and is determined by immunological, genetic, environmental and virological factors [4]. CD4 cell and viral load counts have remained the two strongest correlates and surrogate markers of HIV disease progression regularly used in the clinical setting to monitor the infection [5]. Of the two, CD4 count is a more accurate biomarker of the stage of HIV and is recommended by all guidelines of HIV management [6]. Some studies also argue that CD4 cell count predicts clinical information (event time data) [7] whereas HIV viral load trajectories largely determine the time from initial infection to AIDS: high initial viral load is a marker for rapid progression [8]. Further, many studies report that there is a relationship between these biomarkers, often explaining the disease progression of one biomarker according to the other [9–11].

HIV disease patients progress through normal, mild, advanced and severe clinical stages, and the disease diagnosis can be considered as one of these states. So, instead of modeling of CD4 count without binning into categories, the CD4 count progression should be modeled as a multi-state process, which takes into account viral load dynamics, to explain the development of such diseases. Simultaneous modelling of these two biomarkers may be a better way to capture the complete disease process and progression of HIV/AIDS than individual modelling. It is also essential to understand and predict accurately the dynamic evolution of the disease, which is of particular relevance to physicians who need to distinguish the different types of intermediate events in order to properly adapt treatment plans. Thus, the research focus of the current study is to model viral load dynamics and CD4 cell count progression in an ART-naive cohort.

Previously, a multi-state Markov process modelling of disease progression of HIV/AIDS has been examined by several authors [12–15]. In particular, Binquet et al. [12] estimated the impact of CD8 cell count, weight loss, drug use, gender, viral load and haemoglobin on the progression of HIV/AIDS. Oliveira et al. [14] studied the degrees of chronicity of HIV/AID and went further to examine the impact of covariates; adherence, disease duration, and age on CD4 cell count progression. Recently, Shoko et al. [15] and Chikobvu and Shoko [13], also studied disease progression of HIV/AIDS. However, no previous study has jointly modeled the transition intensity of sequential adversity of the events and longitudinal viral load biomarkers of HIV/AIDS. In addition to that, although the factors related to disease progression of HIV are multiple and complex, very few studies have directly examined the effects of several clinical attributes (ie: white blood cell parameters, RBC parameters, blood chemistry parameters and QoL domain scores) on both viral load trajectories and transitions intensity of sequential events. This study thus gives an insight on presenting a joint multistate model for predicting the clinical progression of HIV infection which takes into account the viral load biomarker, to study several factors that may affect the transition intensities between sequential events.

During the last few decades, some joint models have been developed to study longitudinal biomarker and competing risks using scleroderma lung study data [16, 17]. Han et al. [18] and Cai et al. [19] also provided a joint analysis of longitudinal markers and recurrent events using Epileptic seizure data and Biocard data, respectively. Chi and Ibrahim [20] extended the joint modeling framework to multivariate repeated measurements of longitudinal biomarkers and multivariate survival data using breast cancer data. In contrast, the present work is focusing on joint modeling of longitudinal biomarker and time to transitions between sequential states with application to HIV/AIDS. This is an important aspect that has not been considered in many medical studies particularly in HIV/AIDS cohort studies. This study contains a multi-state model and a linear mixed model, both linked by shared random effects. The goals of joint modelling are to improve inference for the time to transition between multistate events, whilst taking into account the endogenous nature of a biomarker [21]. It also examines the association between the two correlated outcomes [22]. This will lead to an improvement in estimation for a longitudinal biomarker response variable, subject to an informative dropout mechanism that is not of direct interest [23] and will improve inferences as compared to the separate analysis of the two response variables [24].

The goals of this paper are thus threefold. Firstly, we seek to present a joint model of a multistate and longitudinal process, briefly describing previous approaches. Secondly, we seek to apply this model to describe the trajectory of HIV in ART-naive South African patients, so as to aid a deeper understanding of the HIV disease progression process and discover possible factors that influence disease progression. Finally, we will compare the parametric estimator (estimated from the joint multistate model) to nonparametric methods.

## Methods

### Data description

The data is from an ongoing prospective cohort study conducted by the Centre for the AIDS Program of Research in South Africa (CAPRISA) among ART naïve HIV-infected women. The original study, which started in 2004, enrolled a cohort of HIV uninfected women whose age was greater than 18 years with the aim to describe immunologic, clinical and virologic characteristics of HIV-1 disease [25]. In this study, study enrollment was conducted from August 2004 to December 2017. The participant who seroconverted during the HIV uninfected stage of CAPRISA_002 and other CAPRISA prevention and seroincidences trials (including the CAPRISA_004 trials), were enrolled into the Acute HIV Infection phase, and then followed-up during chronic infection and up to ART initiation. Participants were recruited at two sites in KwaZulu-Natal-South Africa, a rural site in Vulindlela and an urban site in the city of Durban. Women without well documented estimated date of HIV infection, and those who did not have at least two follow-up clinical attribute measurements were excluded in this analysis. Finally, 219 participants were included in the study. Further information about the above mentioned ongoing prospective HIV cohort study (CAPRISA_002), including women eligibility criteria and the enrollment procedures were reported in [25–27].

### Variables and measurements

Once HIV diagnosis was confirmed, participants were followed-up for a maximum of 11 years up to cART initiation. CAPRISA initially enrolled HIV-negative (phase I) women into different study cohorts. The women who seroconverted were enrolled into acute infection (i.e. phase II: weekly visits up to 3 months post-infection), then into early infection (i.e. phase III: monthly visits from 3 to 12 months) subsequently into established infection (i.e. phase IV: quarterly visits for more than 12 months) and afterward on cART (phase V). Patients were offered to start cART according to South African (SA) treatment guidelines. SA guidelines for ART eligibility have changed over time during the study period: severe clinical disease or CD4 cell count ≤200 cells/mm3 until 2010; expansion to CD4 cell count ≤350 cells/mm3 for patients with TB and pregnant women from 2010; further to all SA HIV infected patients with CD4 cell count ≤350 cells/mm3 until 2015. In 2013, the WHO recommended treatment at ≤500 cells/mm3, which was then expanded to universal treatment in 2015. South Africa moved to a threshold of ≤500 cells/mm3 in 2016. For the purpose of this study, samples of immunological, virological and clinical attributes (such as viral load, WBC parameters, RBC parameters, blood chemistry parameters, CD4 cell count, etc.) were measured at each visit (phase II-IV). These longitudinal immunologic, virologic and clinical measurements, were recorded for several followed-up visits.

There was a total of 8760 follow-up visits recorded from 219 HIV infected black women with a median age of 25 years (Interquartile range, IQR, 22–30). Of these patients, 9.2% of them were co-infected with TB. Over two-thirds (69.9%) reported having completed grades 11/12 of schooling. The follow-up time of the participants ranged from 0-year to 11.10 years with a first quartile of 0.37 years, a median of 1.50 years and a third quartile of 4.04 years. The median baseline CD4 count of the participants included in the analysis was 519.0 cells/mm3 (IQR 419–655.5 cells/mm3) and the median CD4 counts at ART initiation was 408.0 cells/mm3 (IQR 307.0–587.0 cells/mm3). The log_10_ copies/ml VL count of the participants ranged from 1.47 to 6.81 with a first quartile of 3.84, a median of 4.46 and a third quartile of 5.06.

**Fig. 1:**
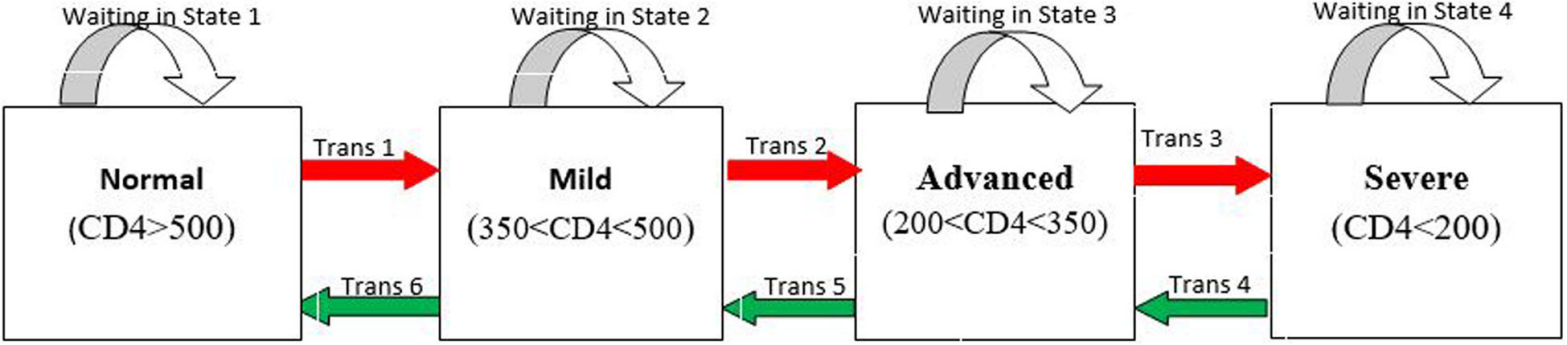
Progressive four-state model based on CD4 counts: Immunological recovery (Green arrows) and Immunological deterioration (red arrows)

The main outcome variables in this current paper are the transition times between multiple states and the longitudinal viral load dynamics (baseline VL and long-term viral load change). In line with the World Health Organization (WHO) immunological classification criteria, which is used to assess the degree of severity of the HIV infection of patients, we have categorized CD4 count. Medical practitioners and health workers also use such WHO classifications to monitor HIV infected patients. These HIV infection stages are defined as no adverse events (CD4 ≥ 500), mild (350 ≤ CD4 ≤ 499), advanced (200 ≤ CD4 ≤ 349) and severe (CD4 < 200) [6]. (See Fig. 1).

**Fig. 2:**
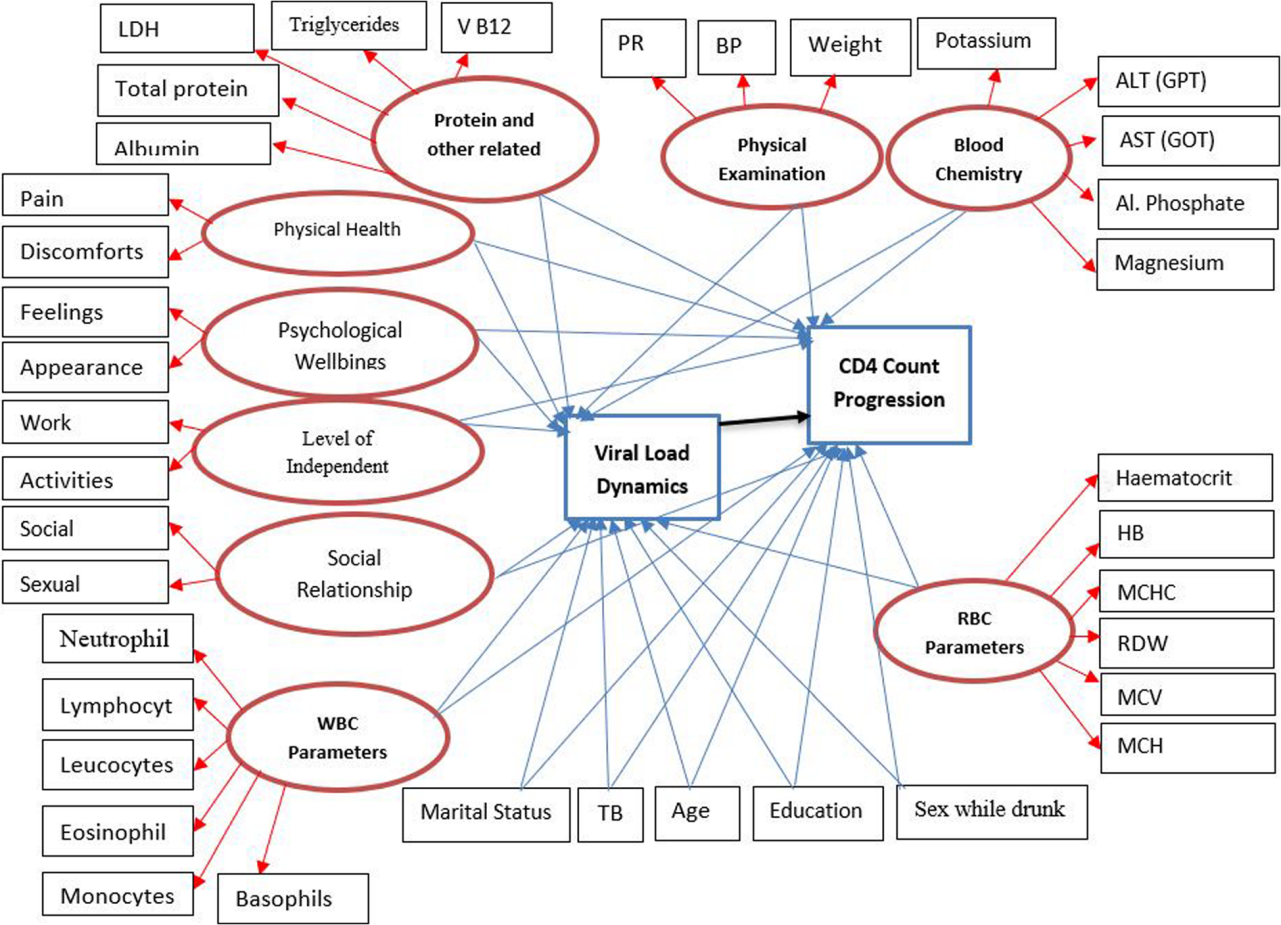
Graphical display of the hypothesized model

The effect of numerous possible factors on the transition times of sequential adverse events and longitudinal viral load biomarkers were evaluated, including (1) demographic variables, (2) risk variables, (3) past opportunistic infections, (4) health-related quality of life domain scores and (5) clinical attributes (see Fig. 2).

### Statistical method

#### Factor analysis

Since our data have a large number of clinical variables, we used exploratory factor analysis in order to group and minimize the number of variables. Factor analysis was done by creating the principal components of the original variables and then creating the eigenvectors. By using the Kaiser-criterions, eigenvectors with eigenvalues greater than 1 were kept [28]. A maximum likelihood extraction method with varimax rotation was used. Factor loadings describe the relationship of each clinical variable with each factor. The Factor loading is considered strong if greater than 0.6, moderate if 0.4–0.6, and weak if less than 0.4 [29]. Each observation was assigned a score for each rotated factor on the basis of the loadings of the subject’s original variable levels. Accordingly, from the 24 clinical variables in the study, we managed to group them in order to create 9 latent variables, defined as granulocytes components, mononuclear components, eosinophils component, RBC component, red blood cell indices, liver abnormality component, electrolyte component, lipid component and protein component. (See Table 1).

**Table 1:** Clinical parameters and Corresponding factor loadings from the rotated factors

WBC parameters	Rotated Factors Loadings		
	1. Granulocytes component	2. Mononuclear component	3. Eosinophils component
Leucocyte	**0.925**	0.282	0.146
Neutrophils	**0.936**	−0.158	0.022
Total lymphocytes	0.226	**0.838**	−0.109
Monocytes	**0.635**	0.417	−0.032
Eosinophils	0.085	0.058	**0.947**
Basophils	−0.035	**0.616**	0.339
RBC Parameters	Rotated Factors Loadings		
	4. Hb and Haematocrit component	5. Red blood cell indices component	
RBC counts	**0.946**	−0.130	
Hb	**0.886**	0.439	
Haematocrit	**0.919**	0.366	
MCV	0.075	**0.953**	
MCH	0.024	**0.825**	
MCHC	0.201	**0.521**	
RDW	−0.382	**−0.592**	
Blood Chemistry	Rotated Factors Loadings		
	6. Liver enzyme abnormality component	7. Electrolyte component	
Chloride	−0.023	**0.455**	
Alkaline	0.174	0.032	
ALT (GPT)	**0.829**	−0.073	
AST (GOT)	**0.967**	−0.122	
Sodium	0.103	**0.994**	
Calcium	−0.020	**0.213**	
Protein and related	Rotated Factors Loadings		
	8. Lipid component	9. Protein component	
Cholesterol	**0.971**	0.027	
LDL	**0.917**	−0.129	
Triglycerides	**0.360**	0.341	
LDH	0.052	**−0.769**	
Total protein	−0.009	**0.670**	

#### Joint multistate model formulation

Let a Markov process {*S*(*t*), *t* ∈ *T*} that has finite space, denoted by ={1, 2, 3, 4}, be a representation of the transition process, where for each patient, a multistate process is observed. Here *T* = [0, *τ*] for *τ* < ∞. This Markov process has an initial probability, denoted by P(*S*(0) = *m*), *m* ∈ *E*, which evolves over time and with a history (*H*_*E*_), which contains the state previously visited, durations and times of transitions [30, 31]. The multi-state process is defined through transition probabilities between two states *m* and *j* relative to the given process history, as:

$$ {P}_{mj}\left(z,t\right)=P\left(S(t)=j|S(z)=m,{H}_E\right)\  for\ m,j\in E\  and\ z,t\in T,z<t. $$

P_m,j_(z,t) thus denotes the transition probability of the patient being in state *j* at time *t*, given that the patient was in state *m* at time *z*.

In the current study, we consider a joint model for the long-term trends of viral load biomarker and transition times into the different adverse effect states based on CD4 cell counts. This model consists of two related submodels: a Markov multistate model for the transition times data and a linear mixed effect model for the longitudinal data (longitudinal measurements of the viral load marker) process, both linked by a function of the shared random effects. HIV-infected ART-naive patients may experience many CD4 cell count fluctuations mostly before ART initiations. This suggests that transition times should be modelled by a multistate process. Figure 1 shows a flow diagram of the multi-state model.

#### Longitudinal submodel

To model the long-term trend of viral load dynamics, we used a linear mixed effect model. Let *y*_*it*_ (*i = 1,2,…,n*; *t = 1,2,…*,*n*_*i*_) be the viral load count of HIV infected patient *i* at follow-up visit time *t*. Under Gaussian assumptions, the longitudinal viral load dynamics *y*_*it*_ is modelled using the following model which is proposed by [32].

$$ {\boldsymbol{y}}_i(t)={\boldsymbol{x}}_i^{\prime}\left(\boldsymbol{t}\right)\boldsymbol{\beta} +{\boldsymbol{z}}_i^{\prime}\left(\boldsymbol{t}\right){\boldsymbol{b}}_i+{\varepsilon}_i\left(\boldsymbol{t}\right)\ \left(\mathbf{1}\right) $$

where ***x***_*i*_(*t*) and ***z***_*i*_(***t***) represents a vector of potentially time-varying covariates corresponding to the vector of fixed effects ***β*** and random effects ***b***_*i*_**,** respectively. The model assumes that random effects are distributed as multivariate normal with mean **0** and covariance matrix **D**. We also assume that the errors are independent and follow a normal distribution, *ε*_*i*_(***t***)***~****N*(0, *σ*), and that ***b***_*i*_ and *ε*_*i*_(***t***) are independent [33]. The normality assumption of longitudinal biomarker measures was checked via the Q-Q plot using the transformed residuals errors based on the fitted joint model [34]. The random-effects represent the effects of each subject on the longitudinal measures that cannot be explained by the observed covariates.

#### Multistate submodel

In this sub-section, we model the transition intensities defined by CD4 cell count which takes into account the viral load biomarker. The standard approach of modelling the event history data [35], in which, for patient *i*, the instantaneous hazard rate of moving to state *j* conditioned on state *m* is defined as follows

$$ {h}_{mj}(t)=\underset{\delta t\to 0}{\lim}\frac{P\left(S\left(t+\delta t\right)=j|S(t)=m\right)}{\delta t}\ (2) $$

$$ {h}_{mj}\left(t;\boldsymbol{X}\right)={h}_{mj,0}(t)\exp \left({\boldsymbol{X}}_{mj}^{\prime }{\boldsymbol{\alpha}}_{mj}\right)\ (3) $$

where *h*_*mj*_ is the transition intensity for *m* = 1, …, 4 and *j* = *m* ± 1 sequential states defined by CD4 counts. *h*_*mj*, 0_(*t*) is the baseline intensity from state *m* to state *j* and can either be left unspecified or modelled parametrically, ***X***_*mj*_ representing a matrix of covariates. ***α***_*mj*_ is the effect of the covariates on the hazard intensity *h*_*mj*_. For this model, if *m* < *j, then a transition from m to j is* defined as an immunological deterioration transition and a transition where *m* > *j* is defined as immunological recovery*.* In the extension of joint modelling, we used a Markov multistate model that takes into account the viral load biomarker through the shared subject-specific random effects **b**_i_. Thus, the instantaneous hazard, for a patient moving from state *m* ∈ E to state *j* ∈ E, at time *t* will be modeled as:

$$ {h}_{mj}\left(t;\boldsymbol{b}\right)={h}_{mj,o}(t)\exp \left({\boldsymbol{X}}_{mj}^{\prime }{\boldsymbol{\alpha}}_{mj}+{\boldsymbol{W}}_{\boldsymbol{mj}}\left(\boldsymbol{b},t\right){\boldsymbol{\gamma}}_{mj}\right).(4) $$

***W***_***mj***_(***b***, *t*) defines a multivariate function that represents the dependence structure between the longitudinal viral load trajectory and multistate CD4 count transitions. We can choose ***W***_***mj***_(***b***, *t*) as the true baseline and slope of the viral load of HIV-infected patients. ***γ***_***mj***_ represents the impact of the longitudinal viral load biomarker on the CD4 cell count progressive form state *m* to state *j*.

#### Association parameters

As one of our objectives is to examine the relationship between transient states defined by CD4 cell count progression and the longitudinal viral load dynamics, we followed the approach adopted by Ferrer et al. [36], where the two sub-models are linked by ***W***_***mj***_(***b***, *t*). This dependence function was the same for all the CD4 cell count transitions and resulted in combining the true baseline and rate of change of the viral load fitted to capture the association between viral load dynamics and the instantaneous hazard rate to transitions between sequential events.

#### Estimation

To estimate the parameters in the shared parameter model, we used maximum likelihood estimation. A joint model can be estimated using the independence between the multi-state and longitudinal processes conditionally on the random effects. So, the likelihood for all the observed longitudinal viral load trajectory (**y**_*i*_) and multistate CD4 count transitions (***S***_*i*_) is given by

$$ \mathcal{L}\left(\boldsymbol{\theta}; \mathbf{y},\boldsymbol{S}\ \right)=\prod \limits_{i=1}^n{\mathcal{L}}_i\left(\boldsymbol{\theta}; {\mathbf{y}}_{\boldsymbol{i}},{\boldsymbol{S}}_i\right) $$

$$ =\prod \limits_{i=1}^n\underset{b_i}{\int }{f}_{VL}\left({\boldsymbol{y}}_i|{\boldsymbol{b}}_i,\boldsymbol{\theta} \right)\ {f}_{CD4}\left({\boldsymbol{S}}_i|{\boldsymbol{b}}_i,\boldsymbol{\uptheta} \right){f}_{\boldsymbol{b}}\left({\boldsymbol{b}}_i|\boldsymbol{\uptheta} \right)d{\boldsymbol{b}}_{\boldsymbol{i}}\ \left(\mathbf{5}\right) $$

***θ =*** (***γ***, ***β***, ***α***, *σ*, **D**)**,**representing all the parameters contained in Eqn. (1) and (4); and the corresponding density functions, is then given by

$$ {f}_{VL}\left({\boldsymbol{y}}_i|{\boldsymbol{b}}_i,\boldsymbol{\theta} \right)=\frac{1}{{\left(2\pi {\sigma}^2\right)}^{{\boldsymbol{n}}_{\boldsymbol{i}}/\mathbf{2}}}\boldsymbol{\exp}\left\{-\frac{\parallel {\boldsymbol{y}}_{\boldsymbol{i}}-{\boldsymbol{x}}_i^{\prime}\boldsymbol{\beta} -{\boldsymbol{z}}_i^{\prime }{\boldsymbol{b}}_i{\parallel}^{\mathbf{2}}}{2\pi {\sigma}^2}\right\} $$

$$ {f}_{CD4}\left({\boldsymbol{S}}_i|{\boldsymbol{b}}_i,\boldsymbol{\uptheta} \right)=\prod \limits_{\boldsymbol{r}=\mathbf{0}}^{\boldsymbol{k}-\mathbf{1}}\left\{{\boldsymbol{P}}_{\boldsymbol{S}\left({\boldsymbol{T}}_{\boldsymbol{r}}\right),\boldsymbol{S}\left({\boldsymbol{T}}_{\boldsymbol{r}+\mathbf{1}}\right)}\Big({T}_r,{T}_{r+1}\left|\boldsymbol{b}\right)\ast {h}_{S\left({T}_r\right),S\left({T}_{r+1}\right)}\Big({T}_{r+1}{\left|\boldsymbol{b}\right)}^{{\boldsymbol{\delta}}_{\boldsymbol{r}+\mathbf{1}}}\ \right\}=\prod \limits_{\boldsymbol{r}=\mathbf{0}}^{\boldsymbol{k}-\mathbf{1}}\left\{\boldsymbol{\exp}\left(-{\int}_{T_r}^{T_{r+1}}\ {h}_{S\left({T}_r\right),S\left({T}_{r+1}\right)}\Big(\boldsymbol{U}\left|\boldsymbol{b}\right) du\right)\ast {h}_{S\left({T}_r\right),S\left({T}_{r+1}\right)}\Big({\boldsymbol{T}}_{\boldsymbol{r}+\mathbf{1}}{\left|\boldsymbol{b}\right)}^{{\boldsymbol{\delta}}_{\boldsymbol{r}+\mathbf{1}}}\ \right\} $$

$$ {f}_{\boldsymbol{b}}\left({\boldsymbol{b}}_i|\boldsymbol{\uptheta} \right)=\frac{\mathbf{1}}{{\left(2\pi \right)}^{\boldsymbol{q}/\mathbf{2}}{\left|\mathbf{D}\right|}^{1/2}}\boldsymbol{\exp}\left\{-\frac{{\boldsymbol{b}}_{\boldsymbol{i}}^{\prime }{\mathbf{D}}^{-\mathbf{1}}{\boldsymbol{b}}_{\boldsymbol{i}}}{\mathbf{2}}\right\} $$

where *f*_*VL*_(***y***_*i*_| ***b***_*i*_, ***θ***) representing the conditional function for the longitudinal viral load trajectory, ||**x**|| represents the Euclidean norm of a vector, *f*_*CD*4_(***S***_*i*_| ***b***_*i*_, **θ**) is the conditional function for the multistate CD4 count transitions, k denotes the number of transitions, ${P}_{\boldsymbol{S}\left({\boldsymbol{T}}_{\boldsymbol{r}}\right),\boldsymbol{S}\left({\boldsymbol{T}}_{\boldsymbol{r}+\mathbf{1}}\right)}\left({T}_r,{T}_{r+1}\right)$ represents the probability of remaining in state *S*(*T*_*r*_) between times *T*_*r*_ and *T*_*r* + 1_**,**${h}_{S\left({T}_r\right),S\left({T}_{r+1}\right)}\left({T}_{r+1}\right)\ \mathrm{denotes}\ \mathrm{the}\ \mathrm{transition}\ \mathrm{intensity}$ to state *S*(*T*_*r* + 1_)**,***δ* denotes transition indicator and *f*_***b***_(***b***_*i*_| **θ**) the joint distribution of the random effects.

Since the dimension of the random effects b_i_ is often high and the density functions of the multi-state Markov process can be highly complicated, evaluation of the above integral (Eqn. 5) can be a major challenge and very intensive. Therefore, for the computations of the above function (eq. 5), a multi-step pseudo-adaptive Gaussian-hermit quadrature rule [24, 36] can be applied to estimate the integrals and to avoids intensive computations of the adaptive quadrature.

The estimation approach was implemented under R (mstate and JM). With JM package initializing the parameters for the longitudinal and multistate sub-models, then, an extended JM() function, called JMstateModel(), which was mainly implemented by Ferrer et al. [36], can carry out the estimation process of the joint model for viral load dynamics and multistate CD4 cell count progression.

## Results

**Fig. 3:**
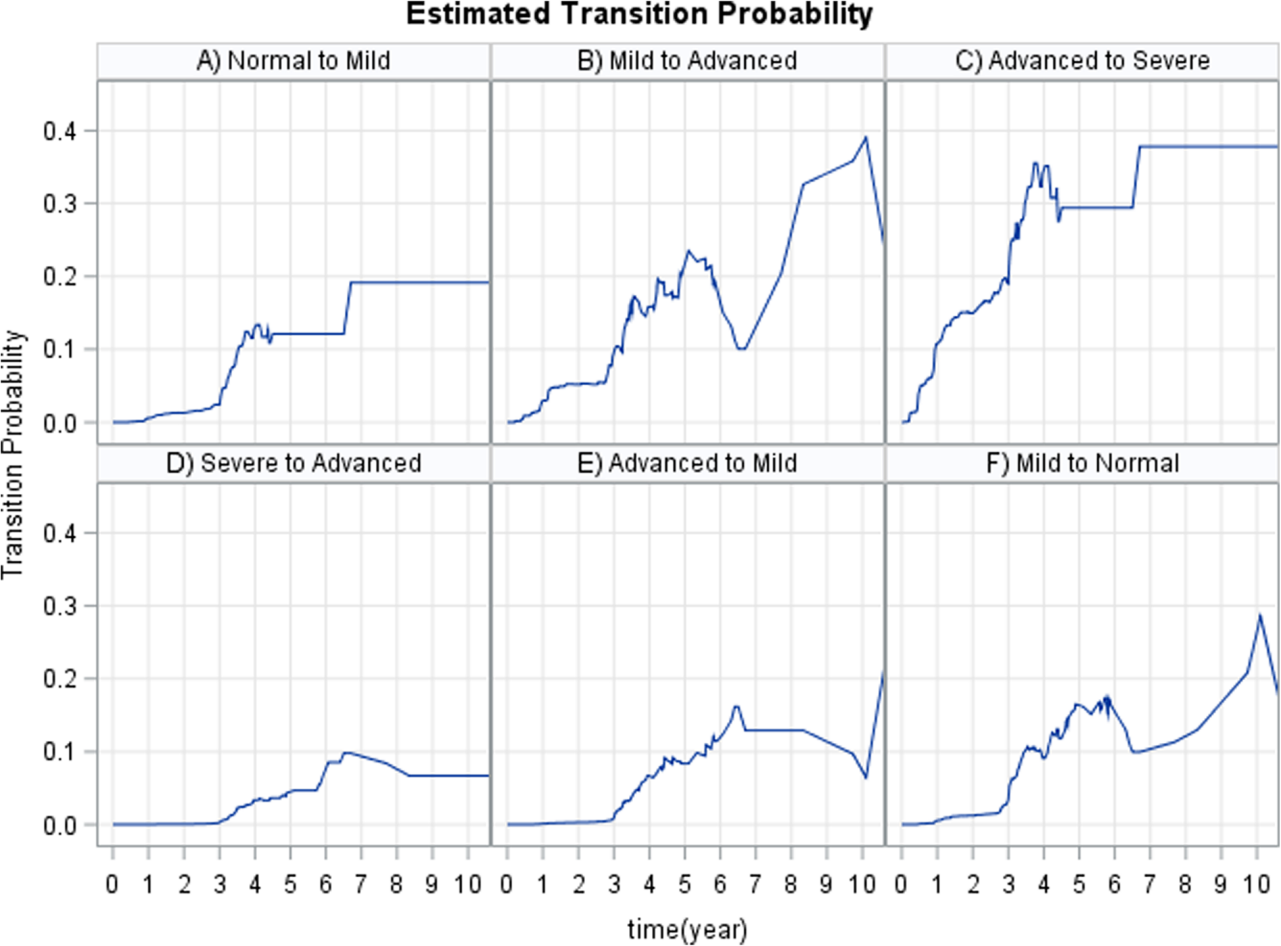
Estimated transition probability using Aalen-Johansen estimator. A) The probability of transition from normal to mild disease state, B) The probability of transition from mild to advance, C) The probability of transition from advance to severe, D) The probability of transition from severe to advance, E) The probability of transition from advance to mild and F) The probability of transition from mild to normal disease state

The completes disease progression situation is visualized and explained by Fig. 1, which shows all possible transitions both immune deterioration (red arrows) and immune recovery (green arrows). In particular, using nonparametric Aalen-Johansen estimation, we calculate the transition probabilities from all starting states to all possible states, between the starting time t = 0 and all event times successively. Thus, Fig. 3 (panels A-C) displays the estimated transition probability from higher CD4 count states to lower CD4 count states (immune deterioration) in HIV-infected women. From Fig. 3 (panels A-C), we note that as the years since enrolment increased, the probability of immune deteriorates (particularly from mild to advanced and advanced to severe stages of the diseases), while the transition probability from lower CD4 count states to higher CD4 count states (immune recovery) not remarkably increasing Fig. 3 (panels D-F). In other words, women who enrolled with a CD4 cell count < 350 (severe and advanced disease stage) had a far smaller chance of immune recovery, and a considerably greater chance of immune deterioration (recurrence) compared to women with higher CD4 cell counts (mild and normal disease stage) at enrollment.

### Results of the joint multistate model

The results of the joint multistate model are presented in Table 2. For longitudinal sub-model results, patients with TB-coinfection (**β** = 0.15; 95%CI: 0.045, 0.249) were significantly associated with a higher baseline viral load, as compared to those who were not co-infected with TB. Similarly, higher educational levels (**β** = − 0.12; 95%CI: − 0.207, − 0.064), higher weight (**β** = − 0.02; 95%CI: − 0.057, − 0.018), higher RBC indices (**β** = − 0.01; 95%CI: − 0.017, − 0.002), higher physical health score (**β** = − 0.01; 95%CI: − 0.026, − 0.003), higher social relationship score (**β** = − 0.12; 95%CI: − 0.027, − 0.001) and patients with stable sex partners (**β** = − 0.12; 95%CI: − 0.207, − 0.064), were significantly associated with lower baseline viral load. When we consider the effects of covariates on the long-term viral load trajectories (the slope), patients with higher educational levels and higher physical health scores were significantly associated with a lower rate of viral load increase over time. Similarly, patients with many sex partners, of younger age (< 20 years) and with higher liver abnormality scores were significantly associated with a bigger increase in viral load in ART-naïve patients.

**Table 2:** Estimates and the 95% confidence intervals for parameters of joint multistate and longitudinal model

Variables	Longitudinal Process	Multi-State Process					
	β (95% CI)	Transition 1: Normal to Mild, β (95% CI)	Transition 2: Mild to Advanced, β (95% CI)	Transition 3: Advanced to Severe, β (95% CI)	Transition 4: Severe to Advanced, β(95% CI)	Transition 5: Advanced to Mild, β (95% CI)	Transition 6: Mild to Normal, β (95% CI)
Intercepts	2.197 (2.016, 2.377)***	**1.41** (0.53, 2.28)*	**1.21** (0.52, 1.91)*	**2.08** (0.71, 3.46)*	**2.46** (0.13, 4.79)*	− 0.01 (− 0.84, 0.83)	**1.71** (0.79, 2.62)*
TB: Yes	0.147 (0.045, 0.249)**	0.61 (− 0.14, 1.37)	0.15 (− 0.43, 0.74)	1.50 (− 0.39, 3.39)	0.41 (− 2.42, 3.23)	− 0.47 (− 1.06, 0.12)	0.98 (− 0.24, 1.72)
Marital status: Married (stable sex partner)	− 0.119 (− 0.170, − 0.068)*	0.44 (− 0.15, 1.03)	− 0.58 (− 1.17, 0.01)	**−1.11** (− 2.15, − 0.06)*	−0.13 (− 1.50, 1.24)	−0.09 (− 0.64, 0.46)	0.05 (− 0.54, 0.64)
Marital status: Many sexual partners	− 0.020 (− 0.117, 0.078)	−0.07 (− 0.40, 0.27)	0.001 (− 0.99, 1.00)	− 0.50 (− 1.23, 0.22)	**−0.61** (− 0.94, − 0.28)*	−0.09 (− 0.47, 0.30)	− 0.02 (− 0.36, 0.32)
Education: 9–10th Grade	− 0.053 (− 0.132, 0.027)	−0.90 (− 1.34, 0.46)	−0.26 (− 0.67, 0.15)	−0.32 (− 1.13, 0.49)	1.02 (− 0.10, 2.15)	0.34 (− 0.13, 0.81)	0.28 (− 0.21, 0.77)
Education: ≥ 11th Grade	−0.136(− 0.207, − 0.064)**	**−1.20** (− 1.66, − 0.74)*	0.03 (− 0.42, 0.48)	0.80 (− 0.06, 1.66)	**0.71** (0.50, 1.91)*	0.19 (− 0.31, 0.68)	**0.59** (0.15, 1.03)*
Age_Cat: < 20 years	0.018(− 0.039, 0.075)	**0.36** (0.01, 0.70)**	0.34 (− 0.03, 0.70)	0.15 (− 0.61, 0.92)	0.55 (− 0.44, 1.53)	0.22 (− 0.18, 0.62)	0.17 (− 0.15, 0.48)
Age_Cat: 21–39 years	− 0.119(− 0.229, − 0.009)*	0.08 (− 0.47, 0.63)	0.11 (− 0.58, 0.79)	−1.36 (− 3.17, 0.45)	1.23 (− 0.58, 3.04)	**1.09** (0.32, 1.85)***	**1.01** (0.48, 1.53)**
Sex while drunk: No	−0.060(− 0.134, 0.015)	0.10 (− 0.31, 0.51)	−0.23 (− 0.67, 0.20)	−0.55 (− 1.29, 0.18)	**1.23** (0.28, 2.17)*	− 0.17 (− 0.58, 0.23)	0.17 (− 0.26, 0.59)
Weight	−0.037(− 0.057, − 0.018)**	**− 0.23** (− 0.33, − 0.12)*	**−0.30** (− 0.42, − 0.17)*	0.03 (− 0.27, 0.33)	0.21 (− 0.24, 0.65)	− 0.03 (− 0.19, 0.13)	−0.03 (− 0.12, 0.07)
Level of independence score	− 0.006(− 0.017, 0.005)	**− 0.48** (− 0.60, − 0.37)*	**−0.11** (− 0.23, 0.01)*	**−0.37** (− 0.65, − 0.09)*	−0.05 (− 0.35, 0.26)	−0.03 (− 0.16, 0.09)	**0.14** (0.03, 0.26)*
Social relationship score	−0.015(− 0.026, − 0.003)*	−0.12 (− 0.24, 0.01)	−0.04 (− 0.16, 0.08)	−0.25 (− 0.49, 0.01)	−0.14 (− 0.44, 0.17)	0.001 (− 0.13, 0.14)	−0.26 (− 0.38, 0.14)
Physical health score	− 0.014(− 0.027, − 0.001)*	**− 0.45** (− 0.64, − 0.27)*	**−0.36** (− 0.54, − 0.17)*	**−0.25** (− 0.61, − 0.11)*	−0.49 (− 0.93, 0.06)	−0.36 (− 0.53, 0.19)	−0.36 (− 0.52, 0.20)
Psychological well-bing score	0.005(− 0.008, 0.018)	0.51 (0.32, 0.70)	0.14 (− 0.04, 0.32)	0.13 (− 0.19, 0.45)	**0.50** (0.11, 0.90)**	0.07 (− 0.10, 0.23)	**0.37** (0.19, 0.55)*
Liver enzymes abnormality component	0.006(−0.002, 0.013)	− 0.08 (− 0.18, 0.03)	−0.04 (− 0.15, 0.08)	0.04 (− 0.15, 0.22)	0.11 (− 0.16, 0.38)	**−0.17** (− 0.30, − 0.05)*	0.04 (− 0.07, 0.16)
RBC indices	−0.009(− 0.017, − 0.002)*	−0.03 (− 0.13, 0.07)	**−0.19** (− 0.30, − 0.07)*	0.04 (− 0.19, 0.26)	−0.07 (− 0.35, 0.21)	−0.08 (− 0.21, 0.04)	**0.16** (0.04, 0.27)*
Granulocytes component	−0.002(− 0.009, 0.006)	−0.08 (− 0.18, 0.02)	**−0.13** (− 0.25, − 0.02)*	−0.05 (− 0.28, 0.18)	0.001 (− 0.31, 0.32)	**0.12** (0.001, 0.25)*	**0.12** (0.01, 0.23)*
Mononuclear component	0.017(−0.009, 0.024)	**− 0.35** (− 0.48, − 0.23)*	**−0.45** (− 0.58, − 0.33)*	**−0.56** (− 0.84, − 0.28)*	0.03 (− 0.23, 0.28)	**0.33** (0.22, 0.45)***	**0.32** (0.21, 0.43)*
**Time Slope**		**Associations between Transitions and Longitudinal Outcomes**					
Visit	−0.007(− 0.015, 0.001)	Transitions based on CD4 count Vs Viral Load	**Coefficient**	**95% CI of β**	***P*-value**		
Weight	0.001(−0.001, 0.001)	Trans 1 vs baseline VL	0.51	(−0.35, 1.37)	0.054		
Psychological well-bing score	0.001(−0.001, 0.002)	Trans 2 vs baseline VL	**−0.33**	**(−0.66, − 0.001)**	0.043*		
Physical health score	−0.004(− 0.008, − 0.001)*	Trans 3 vs baseline VL	0.18	(− 0.11, 0.47)	0.227		
Level of independence score	0.001(−0.001, 0.001)	Trans 4 vs baseline VL	**0.56**	**(0.24, 0.88)**	0.001**		
Social relationship score	0.001(−0.001, 0.002)	Trans 5 vs baseline VL	**1.14**	**(0.75, 1.54)**	0.000***		
Marital Status: Married (stable sex partner)	0.005(−0.003, 0.007)	Trans 6 vs baseline VL	**2.21**	**(1.20, 3.21)**	0.000**		
Marital Status: many sex partners	0.007 (0.003, 0.011)*	Trans 1 vs Time slope VL	0.06	(−0.93, 1.05)	0.909		
RBC indices	−0.01(− 0.09, − 0.002)*	Trans 2 vs Time slope VL	−0.32	(− 1.30, 0.67)	0.528		
TB: Yes	0.002 (0.001, 0.004)*	Trans 3 vs Time slope VL	0.62	(−1.92, 3.16)	0.632		
Sex while drunk: No	0.0001(−0.003, 0.003)	Trans 4 vs Time slope VL	**3.34**	**(0.26, 6.42)**	0.034*		
Age_Cat:< 20	0.008 (0.003, 0.012)*	Trans 5 vs Time slope VL	**2.15**	**(0.69, 3.61)**	0.004**		
Age_Cat: 21–39 years	0.001(−0.001, 0.003)	Trans 6 vs Time slope VL	**1.22**	**(0.05, 2.40)**	0.042*		
Education: 9–10th Grade	0.001(−0.002, 0.004)						
Education: ≥ 11th Grade	−0.001(−0.004, − 0.0003)*						
Liver enzymes abnormality component	0.004 (0.001, 0.01)*						

Keys:- Statistical significance: (*)*P* < 0.05; (**)*P* < 0.01; (***)*P* < 0.001; Reference category: Age [> 40]; Education [≤8 grade]; Marital status [single]; TB [No] and Sex while drunk [Yes]

For the multi-state sub-model results, patients with higher educational levels had statistically significantly increased intensities of transitions from mild to normal (**β** = 0.59; 95%CI: 0.15, 1.03) and severe to advanced (**β** = 0.71; 95%CI: 0.50, 1.91) disease stages, but had reduced the intensities of transitions from normal to mild (**β** = − 1.20; 95%CI: − 1.66, − 0.74) disease stages. Patients with many sex partners had significantly decreased the intensities of transitions from severe to advanced (**β** = − 0.61; 95%CI: − 0.94, − 0.28) disease stages. Middle-aged patients were significantly associated with a higher intensity of transitions from advanced to mild (**β** = 1.09; 95%CI: 0.32, 1.85) and from mild to normal (**β** = 1.01; 95%CI: 0.48, 1.53) disease stages, compared to those patients in the older age group. The psychological wellbeing score had significant effects on severe to advanced (**β** = 0.50; 95%CI: 0.11, 0.90) and mild to normal (**β** = 0.37; 95%CI: 0.19, 0.55) transitions. Patients with high scores of latent variable related RBC indices had significantly increased the intensity of transition from mild to normal (**β** = 0.16; 95%CI: 0.04, 0.27) disease stage, but decreased the intensity of transition from mild to advance (**β** = − 0.19; 95%CI: − 0.30, − 0.07) disease stage. Moreover, having high weight, high physical health score and high level of independent score significantly reduced the intensities of transitions from normal to mild, mild to advanced and advanced to severe disease stages. Furthermore, having a high liver abnormality score significantly reduced the intensities of transitions from advanced to mild disease stage.

Regarding the association parameters between the longitudinal viral load biomarker process (baseline and time slope) and disease progressions of HIV/AIDS, viral load dynamics had a significant effect on the intensities of transitions of HIV/AIDS disease progression. As the baseline viral load of women in the study increased, the likelihood that women transited from advanced to mild (aHR = 0.72 = exp.(− 0.33)) stages of the disease decreased. Similarly, a higher baseline viral load significantly increased the intensities of immunological deterioration transitions. Moreover, as the long-term viral load trajectories of women in the study increased, the likelihood that women transited from normal to mild, mild to advanced and advanced to severe stages of the disease increased.

### Assessment of the fitted model

**Fig. 4:**
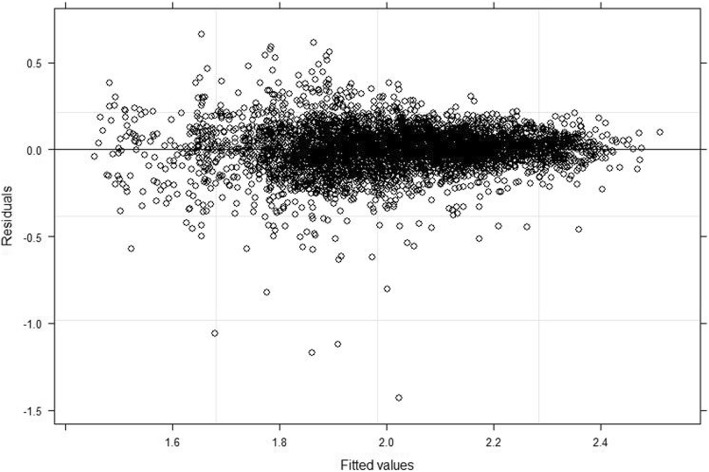
Goodness-of-fit plots for longitudinal viral load biomarker

**Fig. 5:**
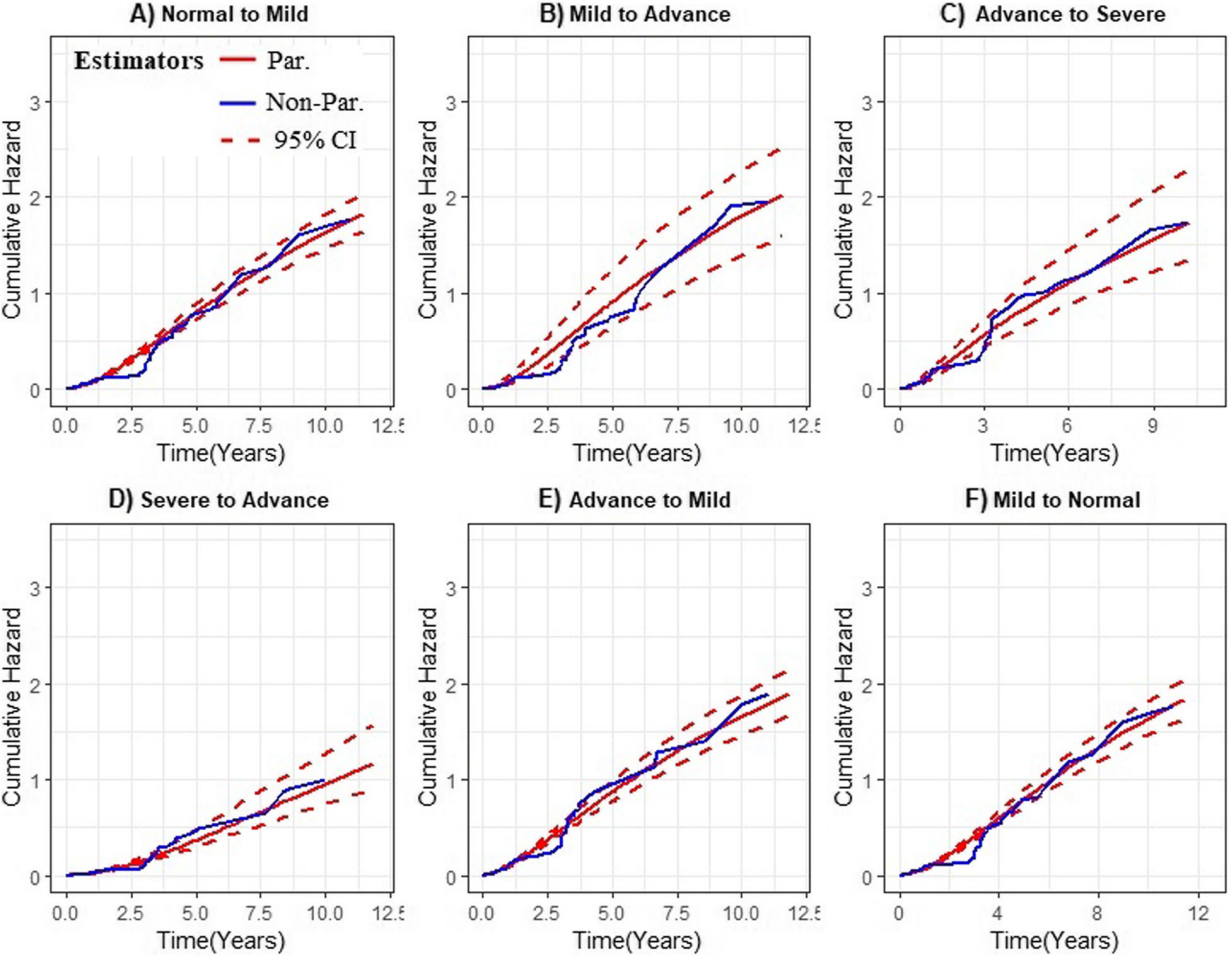
Goodness-of-fit plots. Parametric cumulative hazard estimates (red solid line) and its 95% CI (red dotted line) overlaid on nonparametric Aalen-Johansen estimates (blue solid line)

The estimates of the joint longitudinal viral load biomarker and multistate CD4 cell count transition model were validated by using the graphical methods presented in Figs. 4 and 5. For the mixed effect submodel, the plotted fitted values versus residuals (standardized) of the viral load marker confirmed no heteroscedasticity error (see Fig. 4). For the multistate submodel, the estimates of these multistate models were compared with a nonparametric Aalen-Johansen estimate to assess model fit (as discussed by Ieva et al. [37] and Titman and Sharples [38]). The summary results are presented in Fig. 5 and the six plots showed overall good performances of the joint multistate model in terms of the fit for the transitions cumulative hazard estimate.

## Discussion

In this study, we have presented a joint model for multi-state and longitudinal biomarker data. Such data are common for many medical and clinical studies in monitoring chronic diseases. Simultaneous modelling of this multistate and longitudinal biomarker outcome in joint models offers advantages over a separate model of each outcome, including more clinically meaningful adjusted association parameters, improved predictive accuracy, and more efficient parameter estimates. Also, using this joint multistate model not only an improved inference but also the opportunity for dynamic prediction. Król et al. [39] developed dynamic prediction tools for their recurrent events joint model. Others have also presented dynamic prediction in the context of joint models involving multiple event time data [36, 40, 41]. Hence, in this article the joint model for multistate CD4 cell count progression and longitudinal viral load outcomes provides a complete model of HIV/AIDS disease progression in an ART-naive cohort, which takes into account longitudinal viral load dynamics, to study possible factors that affect time to transition between sequential adverse events of HIV/AIDS. We assumed that the dependency between the transitions time for a given patient is entirely explained by the longitudinal viral load biomarker and the prognostic factors. This assumption could be relaxed by including frailty term in the multistate submodel.

Our results showed that older age has been associated with a higher baseline viral load, which is in agreement with the previous studies [42, 43]. We also found that a faster rise in viral load was significantly associated with young adolescents (age < 20 years) compared to those patients in the older age group (age > 40 years). Having multiple sexual partners significantly increased patients’ viral load throughout the follow-up time. This might be due to patients with high-risk behaviors, such as an increased number of sexual partners, have been associated with depression [44], and patient with chronic depression was significantly associated with an increase in viral load [45]. In other words, viral load has a mediating effect through depression caused by having multiple sexual partners. Moreover, patients with a higher quality of life domain score and higher educational levels were associated with a lower rate of viral load increase over time.

Patients with opportunistic infections and tuberculosis infection, in particular, are associated with increases in HIV viral load [46, 47]. These studies have also been interpreted as tuberculosis accelerating the loss of CD4 count and promote progression from HIV infection to AIDS. Our data add to this literature by showing patients with TB co-infection were associated with a bigger increase in viral load in ART-naïve patients. Contrary to our findings, studies from Nigeria [48] showed that there was no significant relationship between TB co-infection and HIV viral load. Possible explanations for this controversial report might be that data for our study was conducted in a cohort of acutely infected patients and followed up repeatedly over an extended ART-free period. Therefore, patients with active TB should thus be prioritized for viral load monitoring.

Latent variable related to aminotransferase were significantly associated with the change in viral load. Faster rises in the rate of change of viral load over time were observed in patients with higher liver enzymes abnormality. This finding concurs with the prior report [49], which noted that a positive correlation exists between viral load and aminotransferase (ALT and AST). Thus, there is a need to monitor alanine aminotransferase and aspartate aminotransferase levels before initiation of ART, mainly in high-risk patients, to reduce side effect concerns. Furthermore, we found that patients with higher RBC indices scores were associated with a lower rate of viral load increase over time.

The rate of change of immune recovery of a patient with higher education levels increased with greater rates, compared to those with low levels of education. This is supported by the findings of Maurya et al. [50], who found that the level of education significantly affects CD4 cell count and wellness of HIV infected patients. This could be explained as access to education provides a better understanding of hygiene and sanitary practices. Subsequently, proper hygiene and sanitation practices increase the CD4 count. We further observed that patients in the middle-age group experienced higher rates of immunological recovery compared to those patients in the older age group. Consistent with the finding of this study, some studies showed that patients seroconverting at older ages progress more quickly to lower CD4 cell count categories [43, 51]. Furthermore, we found that patients having a high weight significantly increased the intensities of immunological recovery transitions.

The results showed there was a significant positive relationship between QoL domain scores and intensities of recovery of HIV-infected patients. Studies conducted in South Africa [52] and China [53] revealed a similar finding where a better QoL score significantly associated with a higher CD4 count.

Among the different hematological parameters for HIV infected patients, as expected, high scores of latent variable related to total lymphocytes and basophils counts in the blood had significantly reduced intensities of immunological deterioration transitions, a finding that is in accordance with the literature [54], where it was found that *total lymphocytes* and *basophils are* positively and independently associated with CD4 cell count responses. Many studies also suggested that total lymphocytes can adequately serve as a surrogate biomarker for predicting CD4 count progression in resource-limited settings [55–57]. Similar results have been found for latent variable related to neutrophils, monocytes, and leucocytes. Patients having a high score of latent variable related to neutrophils, monocytes and leucocyte count in the blood had significantly reduced intensities of immunological deterioration transitions. This was supported by the previous study [58], where absolute neutrophils and total white cell (leucocyte) counts are independently associated with CD4 cell count responses. Moreover, we found that patients having high RBC parameters score significantly increased the intensities of immunological recovery transitions, but reduced the intensities of immunological deterioration transitions. Furthermore, patients with a high liver abnormality score and with many sex partners showed significantly increased intensities of immunological deterioration transitions.

Viral load biomarker significantly affects the transition intensities of HIV/AIDS disease progression. Having a high baseline viral load significantly decreased the intensities of immunological recovery transitions, but increased the intensities of immunological deterioration transitions. This was supported by previous studies, Farahani et al. [9] and Martinson et al. [59], where higher baseline viral load in early infection has been associated with faster CD4 count decline. Moreover, patients experiencing a higher rate of viral load increase over time have been associated with increased intensities of immunological deterioration transitions. This was supported by a previous study [60], where viral load increases over time were strongly associated with CD4 cell decline. Furthermore, in agreement with the world health organization treatment guidelines [61], which recommends ART in all PLHIV regardless of CD4 cell count, early identification of patients with poor clinical characteristics and initiation of treatment will improve programmatic success and treatment prognosis.

This study has some limitations, including the missing data, which are expected for a study conducted on data collected from patients’ files and when dealing with a long term follow-up period. We did not evaluate model performance with an alternative multistate sub-model, such as a full parametric multistate sub-model (including accelerated failure time models). However, model diagnostics have been performed and the residual and influence diagnostics affirmed no violation of implicit and explicit assumptions in our model. The other limitation is that the study was limited to adult females. Moreover, this joint model did not take into account the simultaneous modeling of many biomarkers and multistate outcomes before and after cART initiation. Bayesian multivariate joint models for analyzing many biomarkers (i.e. viral load and quality of life scores) and multistate outcomes before and after cART initiation, will be our future research work.

## Conclusions

Overall, from a methodological perspective, it can be concluded that the joint multistate model approach provides wide-ranging information about the progression and assists to provide specific dynamic predictions and increasingly precise knowledge of diseases. Joint multistate modelling is necessary to explore the impact of the longitudinal biomarker outcome on the transitions between clinical states. Joint models are also an improvement over separate multisite models because they consider all the longitudinal observations that are predictive of the transition event of interest. Though this research presented the usefulness of the joint multistate model for analyzing the HIV/AIDS cohort data, the approach is applicable to a wide variety of chronic diseases. There is a need for increased research in terms of methods, so hopefully, this article will be helpful applied researchers (for medical research) to familiarize with the method and interpretation of the results therefrom. From a clinical perspective, the findings of this study contribute to extend the survival of the patients and guide clinical interventions.

## Data Availability

The study data is available upon request.

## Abbreviations

PLHIV: People living with HIV
HB: Hemoglobin
V B12: Vitamin B12
BP: Blood pressure
LDH: Lactate dehydrogenase
PR: Pulse rate
RBC: Red blood cells
HRQoL: Health-related quality of life
CAPRISA: Center of the AIDS programme of research in South Africa
OI: *Opportunistic infections\*
*ARV*: Antiretroviral (drug)
ART: Antiretroviral therapy
*AIDS*: Acquired Immune Deficiency Syndrome
RDW: Red cell distribution width
HIV: Human Immunodeficiency Virus
MCH: Mean corpuscular hemoglobin
MCV: Mean corpuscular volume
MCHC: Mean corpuscular hemoglobin concentration
AST: Aspartate aminotransferase
ALT: Alanine aminotransferase
